# Supplementary material for: Facile Preparation of a Bispherical Silver–Carbon Photocatalyst and Its Enhanced Degradation Efficiency of Methylene Blue, Rhodamine B, and Methyl Orange under UV Light
Source: Nanomaterials (Basel). 2022 Nov 10;12(22):3959. doi: 10.3390/nano12223959 (PMC9698814; doi:10.3390/nano12223959)
Supplement: Supplementary file 1 [file nanomaterials-12-03959-s001.zip › nanomaterials-2004229-supplementary.pdf]

Facile Preparation of a Bispherical Silver–Carbon Photocatalyst and Its Enhanced Degradation Efficiency of Methylene Blue, Rhodamine B, and Methyl Orange under UV Light

Md. Akherul Islam [1], Jeasmin Akter [2], Insup Lee [2], Santu Shrestha [2], Anil Pandey [2], Narayan Gyawali [2], Md. Monir Hossain [1,3], Md. Abu Hanif [1], Se Gyu Jang [3,*] and Jae Ryang Hahn [2,4,*]

1 Department of Bioactive Material Sciences, Jeonbuk National University, Jeonju 54896, Korea
2 Department of Chemistry, Research Institute of Physics and Chemistry, Jeonbuk National University, Jeonju 54896, Korea
3 Functional Composite Materials Research Center, Institute of Advanced Composites Materials, Korea Institute of Science and Technology, Wanju, Jeonbuk 55324, Korea
4 Textile Engineering, Chemistry and Science, North Carolina State University 2401 Research Dr., Raleigh, NC 27695-8301, USA
* Correspondence: segyu.jang@kist.re.kr (S.G.J.); jrhahn@jbnu.ac.kr (J.R.H.)

S1. Physicochemical properties of the AgNPs, CNSs, and AgNP–CNS nanocomposites

The optical features of the as-prepared AgNPs, CNSs, and AgNP–CNS nanocomposite were characterized using a UV–vis spectrophotometer (UV-1800, Shimadzu, Japan). The structural morphology, chemical elements, and crystallinity of the products were investigated by X-ray diffraction (XRD, X'Pert MPD, Philips, Netherlands; λ = 1.54 Å, 30 kV, 40 mA), high-resolution transmission electron microscopy (HR-TEM, JEM-2010HR, JEOL, Japan), field-emission scanning electron microscopy (FE-SEM, JSM-64000, JEOL, Japan), energy-dispersive X-ray spectroscopy (EDS, JSM-64000, JEOL, Japan), and Fourier transform infrared (FT-IR) spectroscopy (Perkin Elmer, UK). The surface elements and chemical state of the samples were surveyed by X-ray photoelectron spectroscopy (XPS, K-Alpha, Thermo Scientific, USA). The specific surface area and pore size were analyzed by the Brunauer–Emmett–Teller (BET) and Barrett–Joyner–Halenda (BJH) methods, respectively, using an accelerated surface area and porosimetry system (ASAP 2420 V2.09, Micromeritics, USA). The thermal properties of the samples were characterized using a thermal analysis system (SDT Q600 V20.9 Build 20, TA Instruments, USA). Liquid chromatography–mass spectrometry (LC-MS, LC 1200 Series, Agilent Technologies, USA) was used to identify the degradation products of dye solutions.

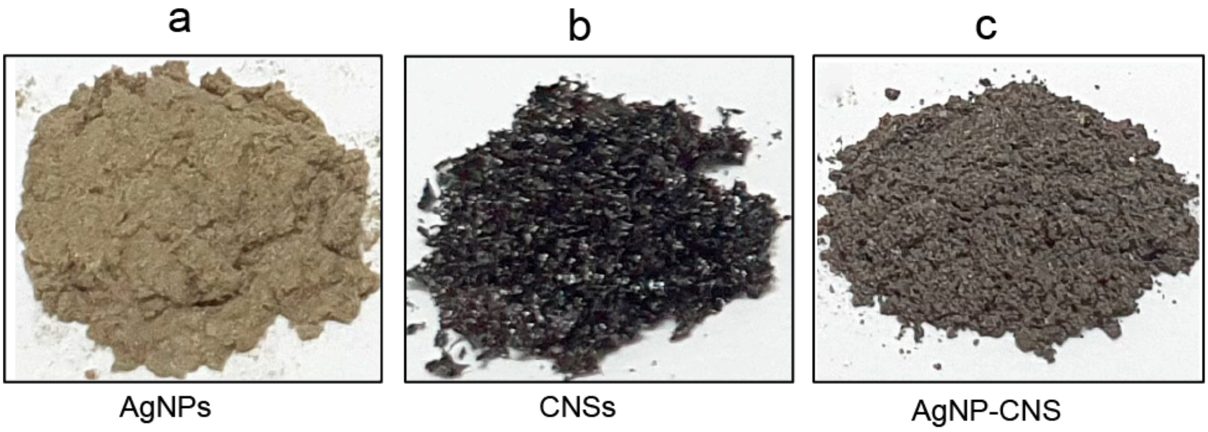

Figure S1. The photos of AgNPs, CNSs, and AgNP–CNS nanocomposite.

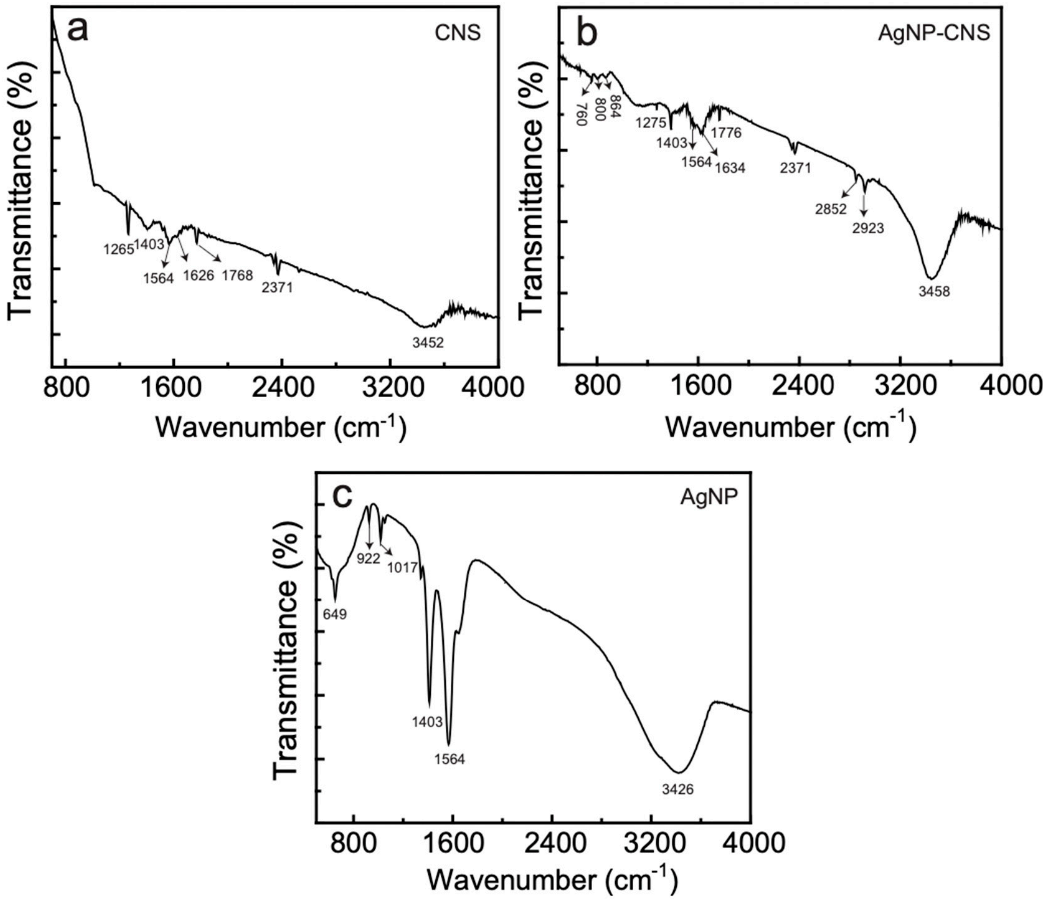

Figure S2. FTIR spectra of (a) AgNPs, (b) CNSs, and (c) the AgNP–CNS nanocomposite.

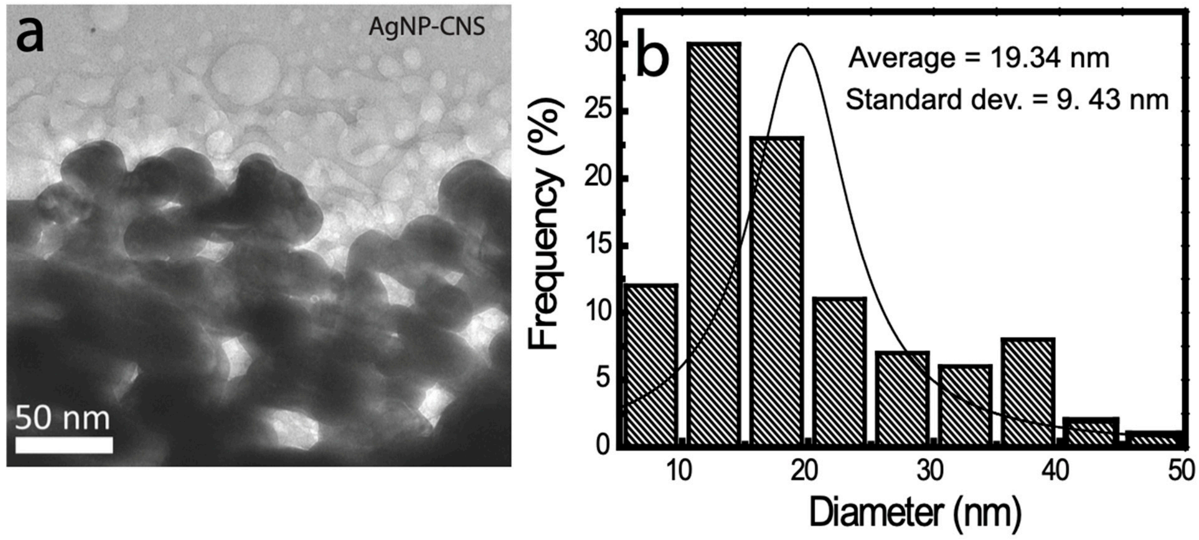

Figure S3. (a) TEM image of AgNP-CNS and (b) a histogram of the diameters.

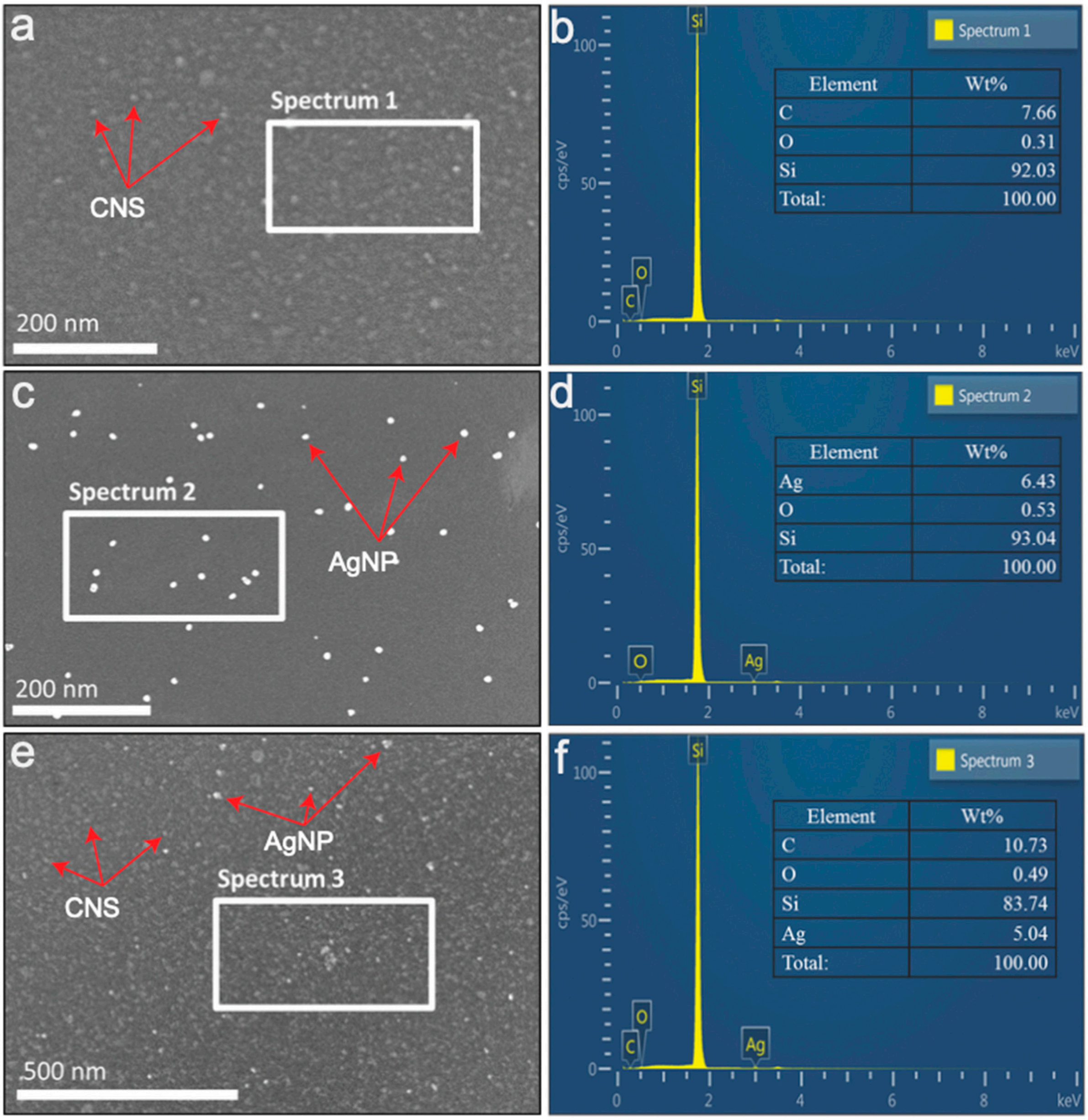

Figure S4. FE-SEM images of (a) CNSs, (c) AgNPs, and (e) the AgNP–CNS nanocomposite. EDX spectra of (b) CNSs, (d) AgNPs, and (f) the AgNP–CNS nanocomposite.

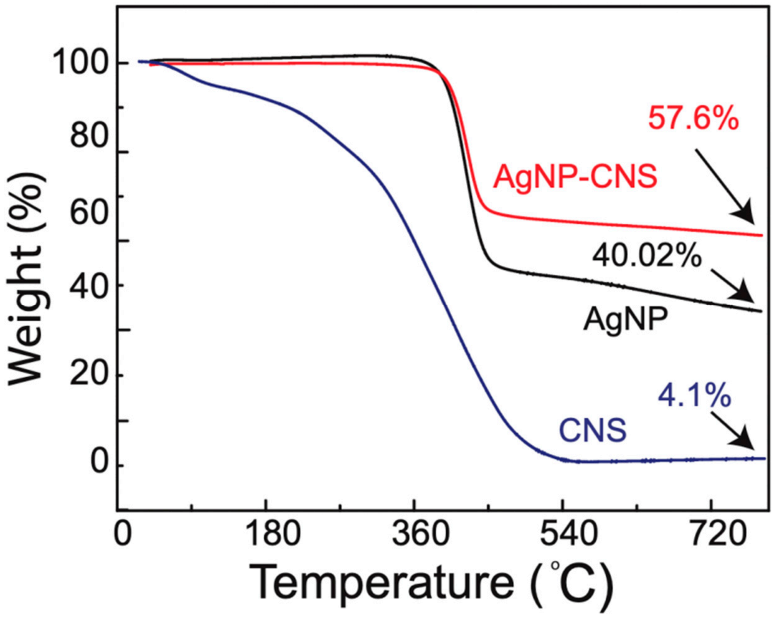

Figure S5. Thermal analysis of the AgNP–CNS nanocomposite, AgNPs, and CNSs.

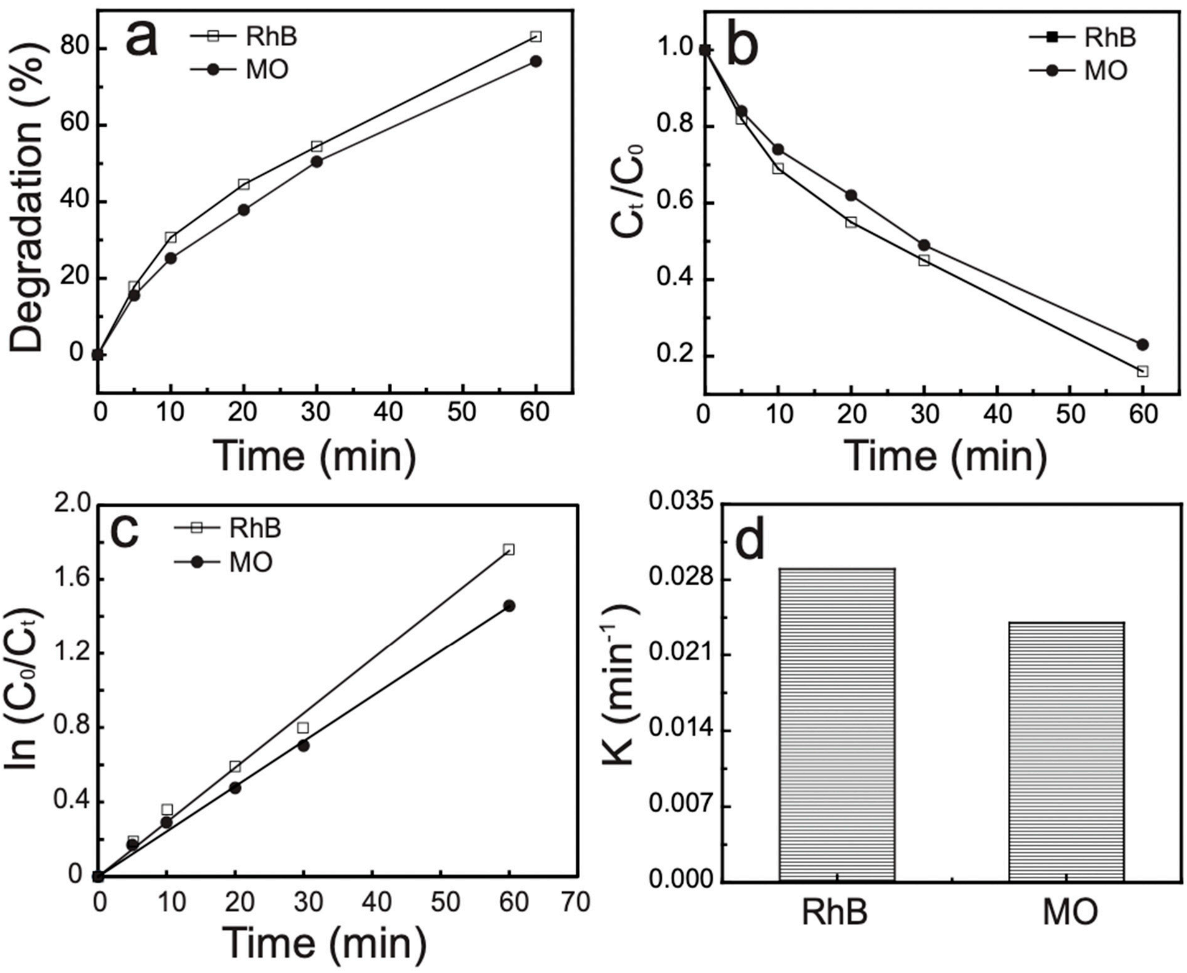

Figure S6. Photocatalytic performance of the AgNP–CNS nanocomposite under UV light: (a) degradation percentage, (b) degradation ratio over time, (c) first-order kinetics analysis for the degradation reactions, and (d) degradation rate constants.

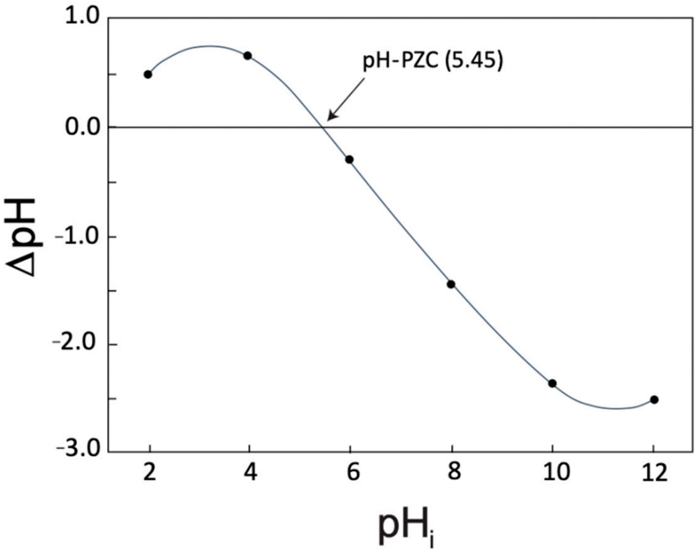

Figure S7. Determination of the point of zero charge of the AgNP–CNS nanocomposite.

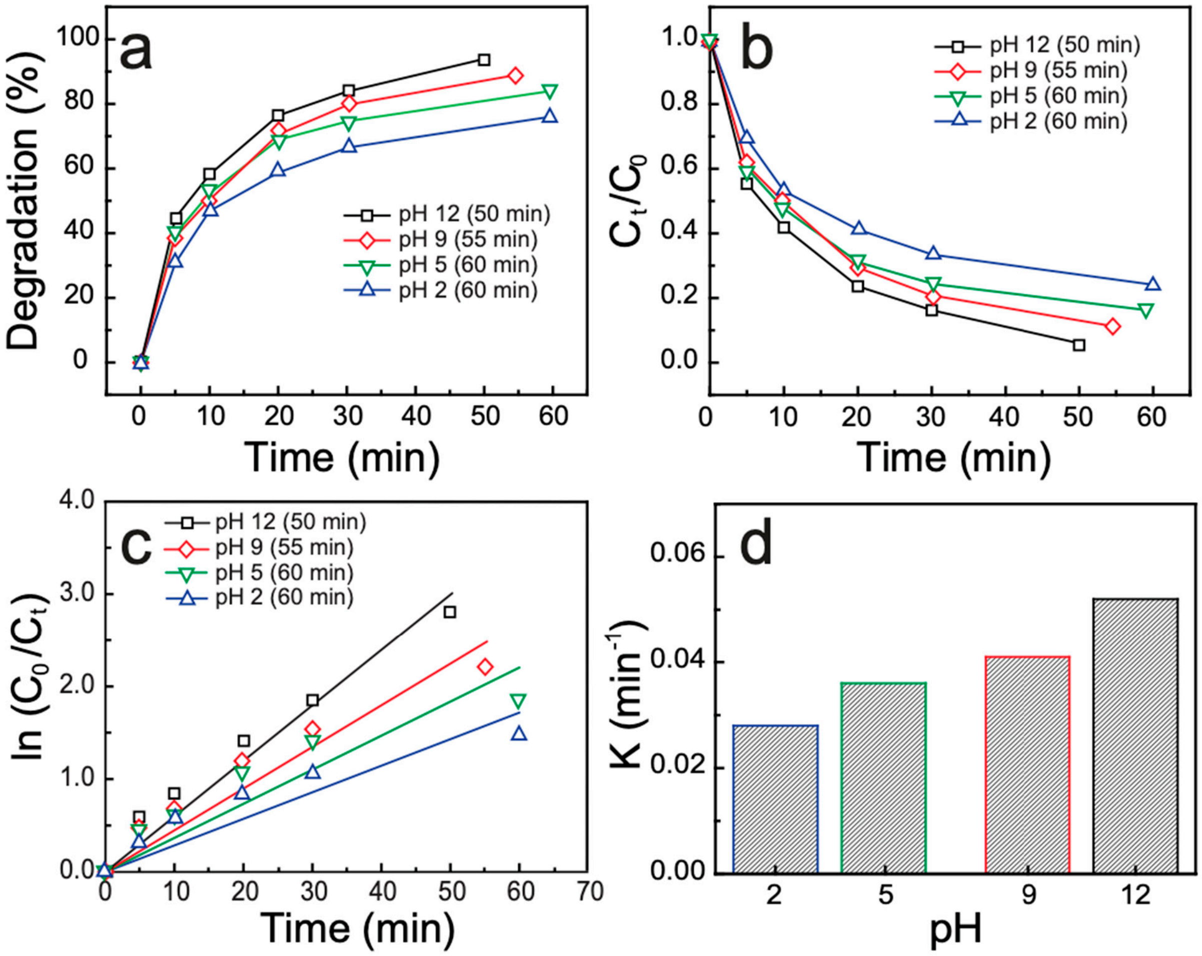

Figure S8. Photocatalytic degradation of MB dye in the presence of the AgNP–CNS nanocomposite under various pH conditions: (a) degradation percentage; photocatalytic performance, as represented by (b) the variation of C_t/C_0 with time, (c) a kinetic plot for dye degradation, and (d) the degradation rate constants of the photocatalytic reaction.

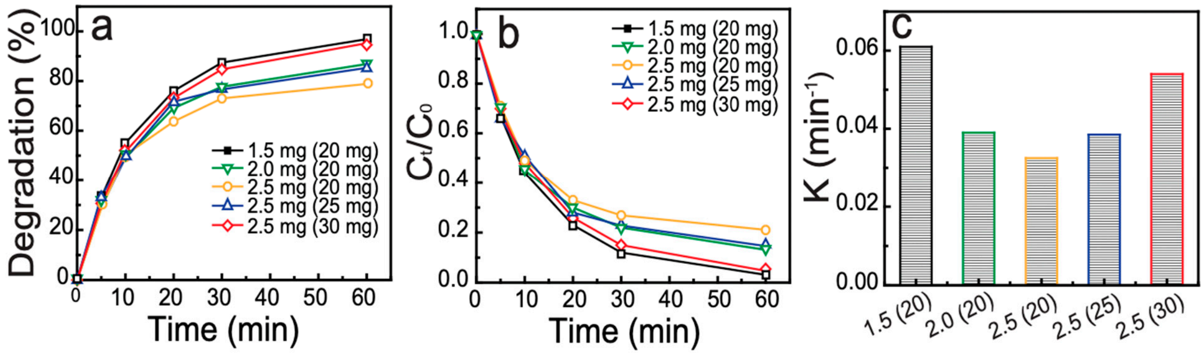

Figure S9. Photocatalytic degradation of MB dye in the presence of the AgNP–CNS nanocomposite under different dose (MB and catalyst) conditions: (a) degradation percentage; photocatalytic performance, as represented by (b) the variation of C_t/C_0 with time and (c) the degradation rate constants of the photocatalytic reaction.

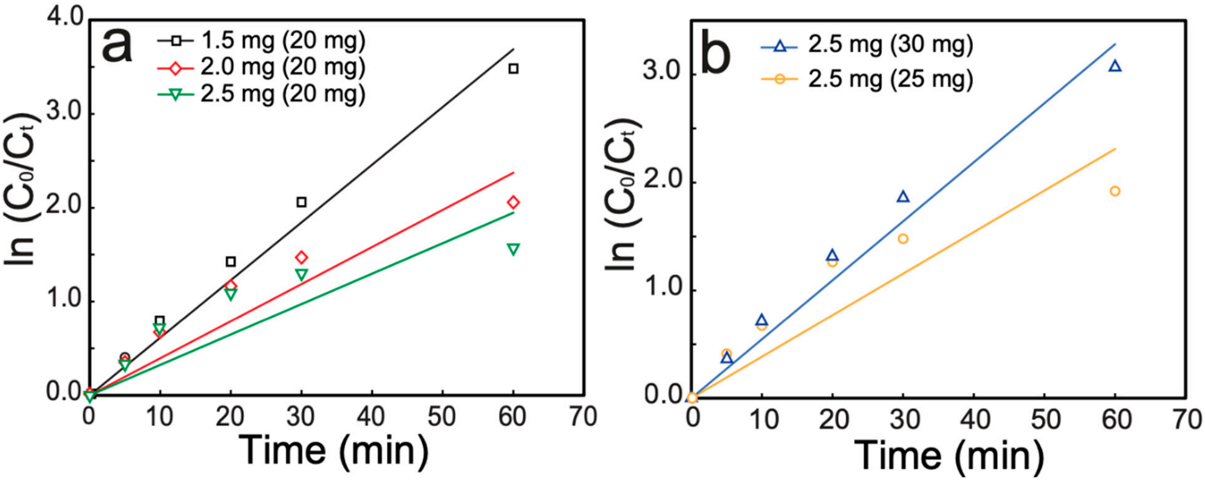

Figure S10. (a, b) The linear relation of ln (C_0/C_t) vs. illumination time confirms the first-order reaction kinetics.

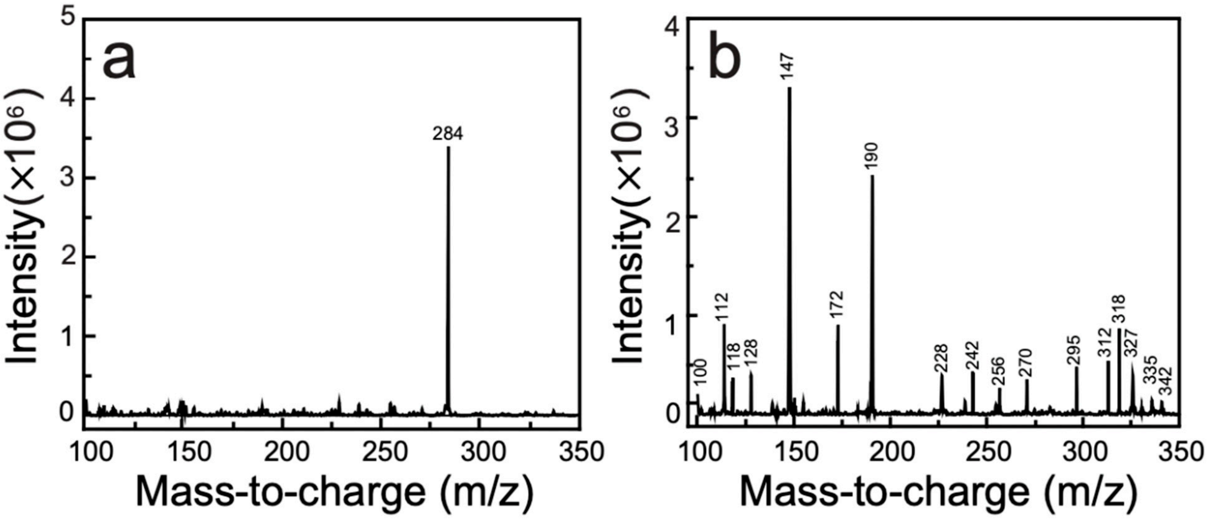

Figure S11. MS data for an MB solution (a) before and (b) after the catalyzed reaction using the AgNP–CNS nanocomposite under UV-light irradiation for 60 min.

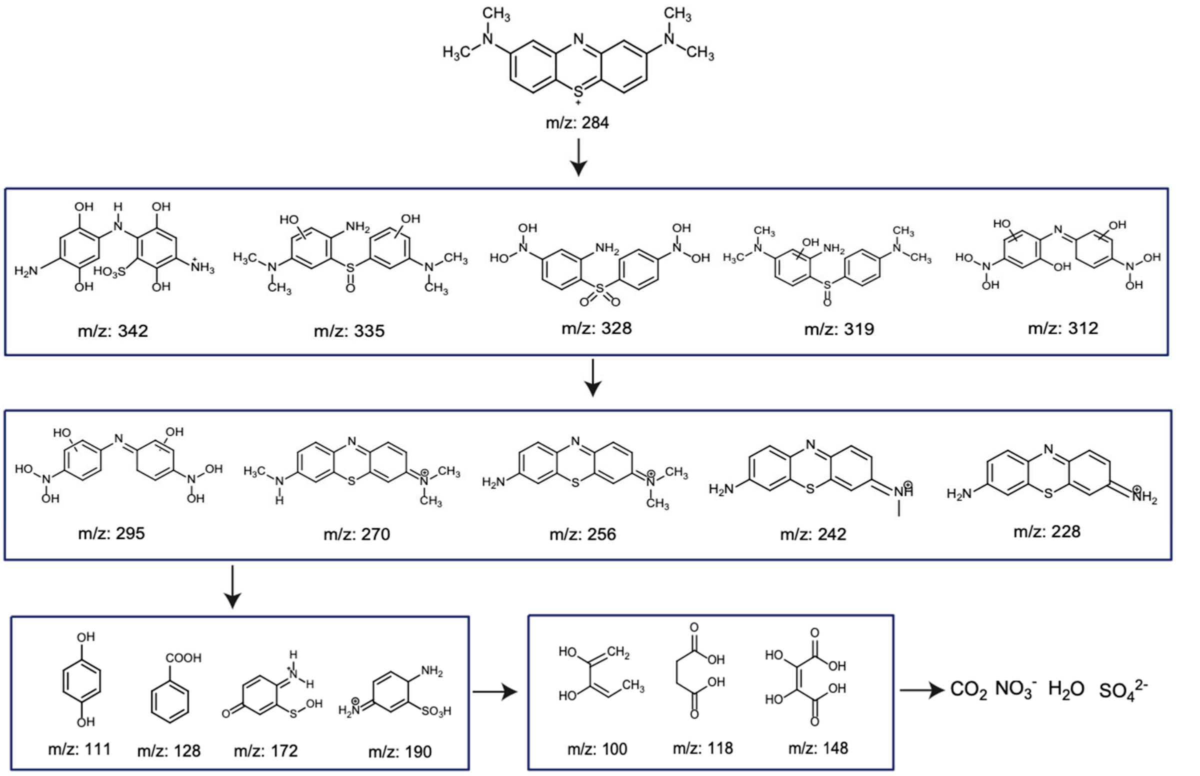

Figure S12. Proposed pathway of degradation of MB using the AgNP–CNS nanocomposite under UV-light irradiation.

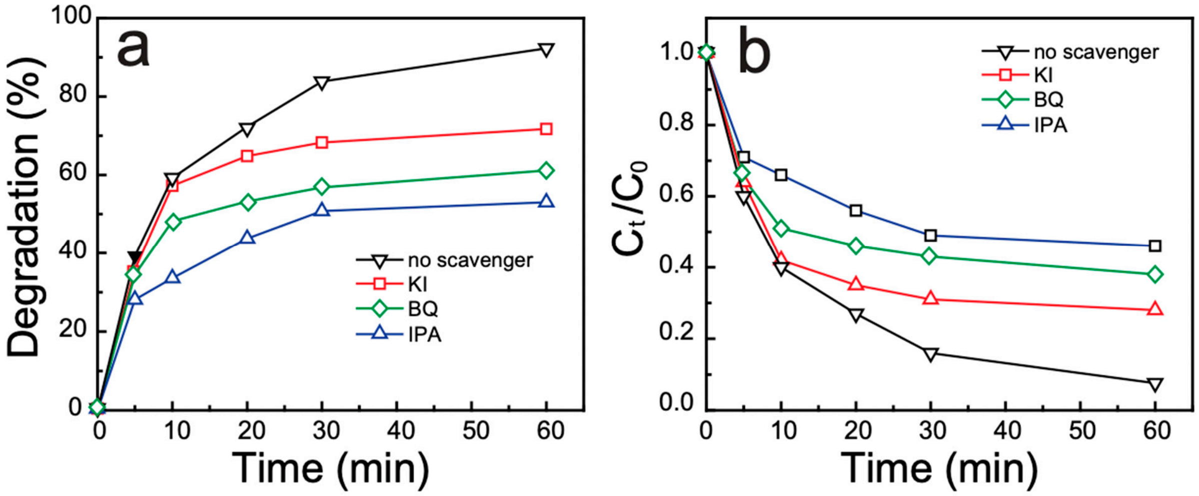

Figure S13. Photocatalytic decomposition of MB evaluated using scavenger agents (KI, IPA, and BQ).
